# Common features in the unfolding and misfolding of PDZ domains and beyond: the modulatory effect of domain swapping and extra-elements

**DOI:** 10.1038/srep19242

**Published:** 2016-01-12

**Authors:** Javier Murciano-Calles, Jofre Güell-Bosch, Sandra Villegas, Jose C. Martinez

**Affiliations:** 1 Departmento de Química Física e Instituto de Biotecnología, Facultad de Ciencias, Universidad de Granada, 18071, Granada, Spain; 2 Department de Bioquímica I Biologia Molecular, Facultat de Biociències, Universitat Autònoma de Barcelona, Bellaterra 08193, Barcelona, Spain

## Abstract

PDZ domains are protein-protein interaction modules sharing the same structural arrangement. To discern whether they display common features in their unfolding/misfolding behaviour we have analyzed in this work the unfolding thermodynamics, together with the misfolding kinetics, of the PDZ fold using three archetypical examples: the second and third PDZ domains of the PSD95 protein and the Erbin PDZ domain. Results showed that all domains passed through a common intermediate, which populated upon unfolding, and that this in turn drove the misfolding towards worm-like fibrillar structures. Thus, the unfolding/misfolding behaviour appears to be shared within these domains. We have also analyzed how this landscape can be modified upon the inclusion of extra-elements, as it is in the nNOS PDZ domain, or the organization of swapped species, as happens in the second PDZ domain of the ZO2 protein. Although the intermediates still formed upon thermal unfolding, the misfolding was prevented to varying degrees.

PDZ domains are protein-protein interaction modules that are highly present in eukaryotic organisms. These domains regulate protein networks by organizing trafficking and signalling in the cell through the localization of receptors, assembly of complexes or adjustment of various sub-cellular structures. Their scaffolding role acts through the specific binding of the C-terminus of the target proteins (usually the last four to nine residues). Members of the PDZ family share a common structure that consists of a five to six β-stranded barrel flanked by two α-helices (Fig. 1A). In spite of their relatively small size (around one hundred amino acids), and apparent simplicity, they perform multiple functions, since a single PDZ domain can bind several partners. Furthermore, additional elements of secondary structure, allosterism, post-translational modifications and domain plasticity can contribute to their functional promiscuity and selectivity^1^,^2^,^3^. Therefore, PDZ domains are useful tools to investigate the relationships between folding and binding^4^.

A universal protein folding scheme for the PDZ domains has been proposed by Gianni, Jemth and co-authors from an extensive kinetic study. Initially, they studied five different PDZ domains and detected a common on-pathway intermediate that separated two transition states, whose positions were practically identical in the reaction coordinate^5^. However, in a more recent study of two additional examples, they described a mechanism with two intermediates, which they then generalized to all cases^6^.

In the present work, we have analysed instead the unfolding equilibrium and subsequent misfolding upon thermal denaturation. Previously, we described the unfolding thermodynamics of the third PDZ domain of the PSD95 protein (PSD95-PDZ3^302–392^)^7^, the only PDZ domain studied up to date in this regard. Results showed that the domain may pass through an intermediate state, prone to reversibly self-associate as a trimer, which drives to misfolding towards fibril structures in an irreversible manner. Now, we have chosen two other prototypical examples, the PDZ domain of Erbin (Erbin-PDZ) and the second PDZ domain of the PSD95 protein (PSD95-PDZ2), to decipher whether that unfolding/misfolding behaviour is a common attribute of these domains.

## Results

### Unfolding and misfolding of the PDZ arrangement

Equivalent to the previously described results for PSD95-PDZ3^302–392^, differential scanning calorimetry (DSC) traces show two calorimetric transitions (Fig. 2), indicating the existence of at least three macroscopic states upon unfolding and thus confirming the presence of an intermediate state. Thus, upon heating, the first transition will represent the heat evolution from the native to the intermediate state, and the second will represent the heat from the intermediate to the unfolded state.

In addition, both endotherms separate from each other along the T axis as the protein concentration is increased in the experiments, suggesting that some association/dissociation processes are taking place. Concretely, the transition at lower temperatures indicates an association of the intermediate state coupled to the unfolding process, since the endotherm moves towards lower temperatures as the protein concentration increases. The second transition, which moves at the contrary, indicates a dissociation of the intermediate when it unfolds. This experimental behaviour has already been deeply explained in the case of PSD95-PDZ3^302–392 ^7.

For Erbin-PDZ and PSD95-PDZ2 reversibility was found to be at least 20–30% for the first transition. In our previous work with PSD95-PDZ3^302–392 ^7 we carried out a systematic study to discard the influence of irreversible processes coming from misfolding on the experimental DSC traces. Thus, we performed some measurements at different heating rates under identical experimental conditions and found a slight displacement of the traces that shift towards high temperatures when the heating rate increased. Nevertheless, we did not observe changes in shape or area, which indicates that the thermodynamic information derived from these traces will not be too much affected, apart from experimental errors, by such kinetic processes. In the cases of Erbin-PDZ and PSD95-PDZ2 we found qualitatively the same behaviour.

The simplest thermodynamic model that may explain this unfolding behaviour (N1⇄/n I_n_⇄)^8^ was unable to fit the data in all cases, unless we introduced an additional stage in the unfolding scheme where a monomeric intermediate species can populate upon unfolding and is subsequently associated^7^:


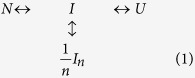


A mathematical description of the model and of the fitting procedure can be found in the Supplementary Information. The best fitting of the two datasets based on this model is shown as black-solid lines in Fig. 2. The quality of the fittings (R^2^ = 0.996 for Erbin-PDZ and R^2^ = 0.998 for PSD95-PDZ2) very likely indicates that both, Erbin-PDZ and PSD95-PDZ2 domains, follow the same unfolding scheme as PSD95-PDZ3^302–392^ and, consequently, that a relatively stable intermediate populates upon unfolding, both as a monomer (I) and also as a self-associated species (I_n_). The stoichiometry of such an association process should be n = 3, since we performed an initial analysis that left this parameter free and floating. The best fittings corresponded to n = 2.8 and n = 3.2 for Erbin-PDZ and PSD95-PDZ2 respectively. PSD95-PDZ3^302–392^ showed similar results with n = 2.8 7. Thus, we can conclude that a trimer is the most likely species of the associated intermediate in all cases. The thermodynamic parameters derived from these analyses are collected in Supplementary Table S1.

To characterize the intermediate at a structural/conformational level, we used Fourier-transform infrared spectroscopy (FTIR) to detect conformational changes in the secondary structures. We recorded the amide I’ spectra of the protein solution at the highest concentration used in DSC experiments (700–800 μM), and at temperatures of either 25 °C or 60 °C wherein the most populated species are the native state and the intermediate, respectively (Supplementary Fig. S1). Our measurement at 60 °C was recorded after a one-day incubation time to allow the supramolecular structures to gain certain stability and also to be able to visualize them by transmission electron microscopy (TEM; see later). Figure 3 shows bar charts displaying the area of the main bands after deconvolution of the amide I’ band for the different PDZ domains.

The spectra at 25 °C, which represent the native states of Erbin-PDZ, PSD95-PDZ2 and PSD95-PDZ3^302–392^, are very similar, the main components being those signifying the flexible and the stable native β-sheets. There is however a difference concerning the aggregation components, namely, the appearance of the amyloid component (1612 cm^−1^) in both the Erbin-PDZ and PSD95-PDZ2 spectra, in addition to the worm-like component (1620 cm^−1^). At 25 °C these bands reflect the tendency to aggregation which already has been described for PSD95-PDZ3^302–392 ^7. In any case, the area of both components is small at this temperature, being the amyloid component even lower than that for the worm-like, and they are quantitatively comparable in the three examples.

Upon heating at 60 °C (Fig. 3), a huge increase in the area of the components corresponding to aggregates (1620 and 1612 cm^−1^ bands) occurs for all three domains. This is apparently generated at the cost of the rest of the bands arising from the native secondary structures, which decrease, thus indicating a structural change wherein a compact, aggregated, β-sheet predominates. These aggregates organize as worm-like fibrils in all three cases as seen by transmission electron microscopy (TEM; Supplementary Fig. S2). Thioflavine T (ThT) fluorescence kinetic experiments at 60 °C constitute a convenient test to distinguish between amyloid and worm-like fibrils, since the former describe sigmoid curves initiated by a lag-phase, whereas the latter follow non-cooperative exponential kinetics without such a lag-phase^9^. As shown in Supplementary Fig. S3, the latter is the case for the three PDZ domains, and thus concurs with the results obtained by TEM.

Consequently, we may say that the PDZ fold shares an organized intermediate, structurally well-conserved, which drives to misfolding through the formation of worm-like fibrils.

### Modulation of the unfolding behaviour by extra-elements and domain swapping

As it is well known, PDZ domains are contained in multi-modular proteins and many of these domains utilize amino acid segments beyond their canonical fold to modulate their functional abilities, which has been noted in literature as ‘extended PDZ domains’^10^. To elucidate in detail how subtle differences in the arrangement of the PDZ domain may affect unfolding and misfolding pathways and, thus, define more details of their unfolding landscape, we selected some PDZ examples which present such particular structural features, as the insertion of extra-elements or the organization of swapped species.

**Figure 1:**
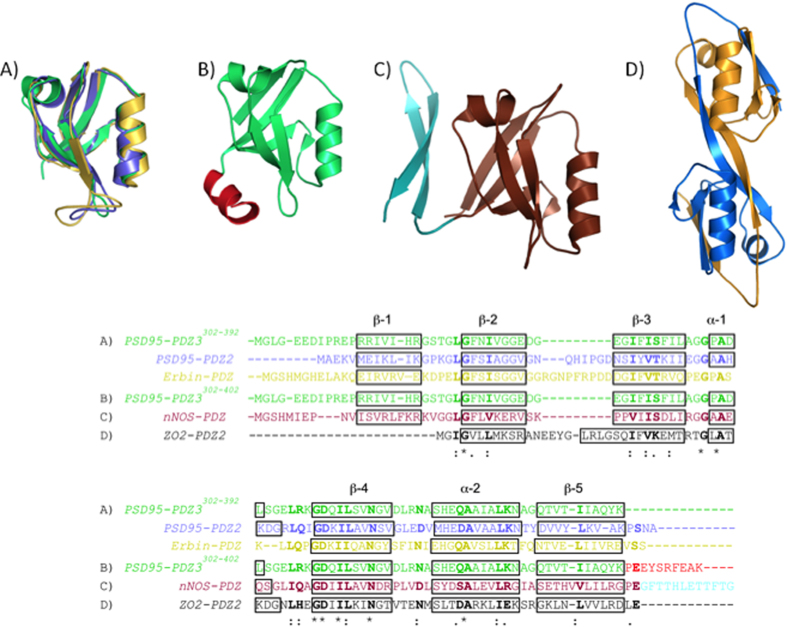
The crystal structures and sequence alignment of the five PDZ domains under study. In the upper panel the following crystal structures are represented (**A)** Superposition of Erbin-PDZ, PSD95-PDZ2 and PSD95-PDZ3^302–392^ (**B)** PSD95-PDZ3^302–402^ (**C)** nNOS-PDZ; and (**D)** ZO2-PDZ2. The extra-elements and swapping phenomena have been distinguished in different colours in the figure. In the lower panel, the sequences correspond to the crystal structures. Symbols under sequences: (*) identify identical residues, (:) report for residues of equal nature, and (.) recognize roughly similar residues.

We have already described elsewhere how the presence of a third α-helix at the C-terminus of PSD95-PDZ3^302–392^, identified here as PSD95-PDZ3^302–402^ (Fig. 1B), prevents somewhat the formation of the associated intermediate, which becomes less populated under the same experimental conditions. It also slows down misfolding, giving rise to supramacromolecular species of a different nature, namely annular structures and worm-like fibrils^8^. Moreover, and opposite to the shorter PSD95-PDZ3^302–392^, these aggregated species can revert to the native state upon cooling and/or diluting the samples, which demonstrates their reversible nature. This C-terminal α-helix, which is the link between the third PDZ domain and the following SH3 domain of the PSD95 protein, also has important consequences in the binding features of the domain^11^,^12^.

**Figure 2:**
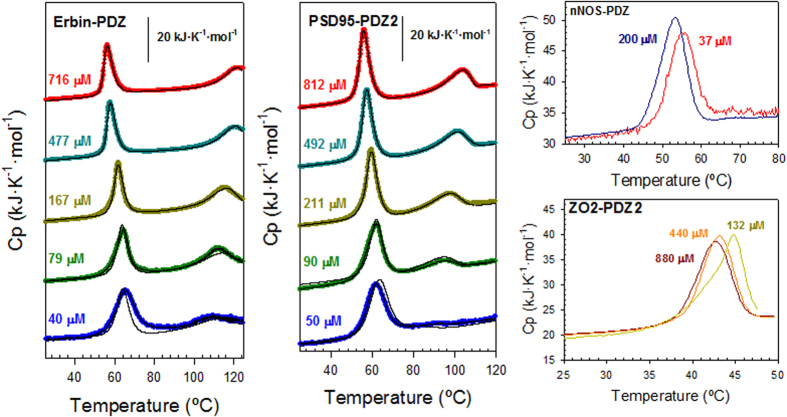
Differential scanning calorimetry (DSC) experiments at different protein concentrations. In the Erbin-PDZ and PSD95-PDZ2 panels, the experimental data are represented by circles with different colours for the different protein concentrations assayed. The black lines correspond to the best fitting results according to the model of equation (1), using n = 3. In nNOS-PDZ and ZO2-PDZ2 panels, experimental data are represented by lines, also using different colours for the different protein concentrations. Experimental conditions were 50 mM phosphate pH 7.5 in all experiments.

**Figure 3:**
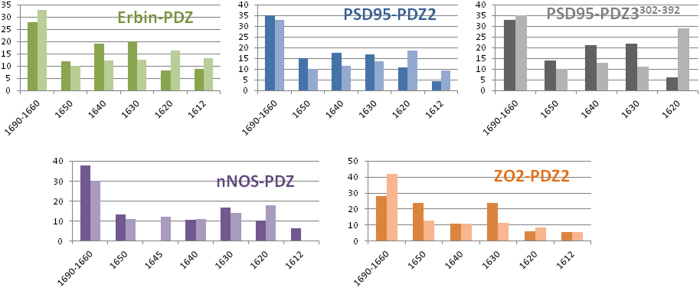
Results of the Fourier-transform infrared spectroscopy (FTIR) spectra analysis of the five PDZ domains under study. Bars represent the relative % of the areas of the main bands after deconvolution of the spectrum. The bands within the 1690–1660 cm^−1^ have been grouped, since they correspond to irregular secondary structures (β-turns and loops) and to the negligible high-frequency component of β-sheets; the one at 1650 cm^−1^ corresponds to α-helices; the 1645 cm^−1^ band to random coil; the 1640 cm^−1^ band to flexible β-sheets (as described in^26^); the 1630 cm^−1^ band to well-organized β-sheets; the 1620 cm^−1^ band to worm-like fibrils; and the 1612 cm^−1^ band to amyloids. The darkest and lightest bars come from the spectra at 25 °C and 60 °C, respectively. Experimental conditions were 50 mM phosphate pH 7.5.

In addition to this previously described example, we chose the nNOS-PDZ domain, which contains an extra β-hairpin in its C-terminus (Fig. 1C). This extra-element interacts with the binding groove of other PDZ domains (PSD95-PDZ2 and syntrophin PDZ) in a non-canonical manner known as “intein mode”, in which no C-terminal free-carboxylate groups participate in the interaction^13^,^14^,^15^. As can be seen in Fig. 2, the presence of such an extra β-hairpin affects the DSC traces; only one transition is detected and the main distinct feature is the almost complete irreversibility of the process. A similar behaviour was found with PSD95-PDZ3^302–415^, a construct that includes the third α-helix and a following β-hairpin at the C-terminus of the PDZ arrangement (Supplementary Fig. S4)^16^,^17^. Remarkably, in both cases, the single remaining transition keeps the behaviour with respect to protein concentration previously described, as seen in Fig. 2 for nNOS-PDZ. These features clearly reveal that the associated intermediate state remains in this example as well. At 60 °C, on the other hand, we observed some differences between the FTIR spectra of nNOS-PDZ and the archetypical PDZ domains described above (Fig. 3): *i)* regular secondary structures (α-helices, flexible and stable native β-sheets) remain more or less unaltered upon heating; *ii)* the random coil component (1645 cm^−1^) splits from that of the flexible β-sheet (1640 cm^−1^); and *iii)* the amyloid band (1612 cm^−1^) disappears to the same extent in which that of the worm-like structure (1620 cm^−1^) increases, so that the total area for both bands reporting for aggregates remains the same. Thus, an increase occurs in the random coil component rather than in the aggregation component observed in the examples with strict PDZ fold. Therefore, amorphous aggregation is competing with fibrilation advantageously enough to achieve that the worm-like fibrils morphology is not as evident as with the previous examples (Fig. S2), despite the fact that non-exponential ThT-kinetics are maintained (Fig. S3).

In conclusion, we can affirm from both examples, PSD95-PDZ3^302–402^ and nNOS-PDZ, that the extra-elements do not appear to change the main unfolding characteristics, as the occurrence of a partially-folded self-associated intermediate, but are clearly modifying misfolding, thus revealing that these extra-segments not only influence binding, but also the folding landscape of the domains.

To complete our investigation using natural-PDZ structure variants, we chose the second PDZ domain of ZO2 (ZO2-PDZ2), as it is well known for organizing swapped structures, in which a dimer is arranged by the inter-exchange of the β2-β3 hairpin within the two molecules (Fig. 1D). Again, the irreversibility of the unfolding process exhibits a single transition that moves towards lower temperatures with increasing protein concentration (Fig. 2), indicating that an association process of the intermediate species that populated after this first transition is taking place. The fully irreversible character of the thermal unfolding, as in the case of nNOS-PDZ, precluded any thermodynamic analysis. FTIR experiments showed no significant changes, with respect to temperature, of the bands for amyloid and worm-like fibril aggregates (Fig. 3), thus indicating that the misfolding route is avoided for this swapped arrangement. This also agrees with the fact that only amorphous aggregates are visible by TEM (Supplementary Fig. S2). In fact, the bands for both α-helices (1650 cm^−1^) and stable β-sheets (1630 cm^−1^) decrease, whereas those for the irregular secondary structures (1690–1660 cm^−1^) increase.

## Discussion

**Figure 4:**
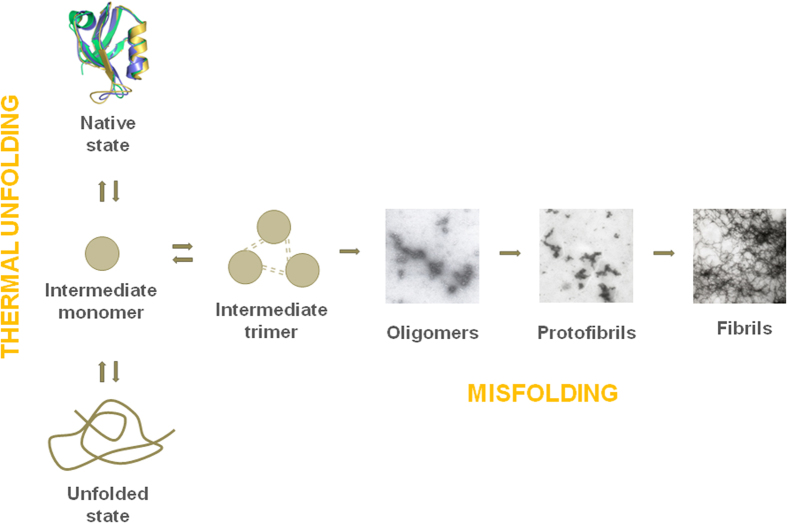
The unfolding landscape of the studied PDZ domains sharing the canonical PDZ fold.

Overall, our results show a common thermodynamic pattern in the unfolding and misfolding routes of the PDZ fold (Fig. 4), where minor structural differences change appreciably the behaviour.

We believe that the selected examples may be representative of the approximately 270-member collection of human PDZ domains, since this domain family has been shown to have a well-conserved structural arrangement. It might be likely that other examples would have similar behaviour, as has been suggested from the previous kinetic studies^5^,^6^ and as also happens within other modular domain families like the SH3 domains^18^,^19^,^20^,^21^. Furthermore, some particular structural features found in these domains, as the inclusion of extra-structural elements at the C-terminus or swapping dimerization, have also been explored. In addition, the selected PDZ domains for this study comprise different specificity classes from the ones defined for these domains^22^. PSD95-PDZ2, PSD95-PDZ3 and Erbin-PDZ have a prototypical binding mode in PDZ domains and belong to class I and II (according to the original classification). The nNOS-PDZ domain may interact both with C-terminal extremes of different targets, defined as specificity class III^23^, or without involving the free carboxylate, defined as “intein mode”, with PSD95-PDZ2, as has been mentioned above. The ZO2-PDZ2 domain has been noted as a binder to lipids^24^. Thus, with these examples we have covered as well almost all the possible binding modes of these domains^25^.

In summary, despite structural and functional differences among the selected examples, the equilibrium unfolding of the PDZ domains appears to be well preserved (Fig. 4). In all cases, associated intermediate species seem to populate upon thermal unfolding and, in the cases where reversibility exists, a thermodynamic analysis can be done by using the unfolding model of equation (1). The trimeric nature of these self-associated intermediates has also been derived from DSC model. The intrinsic low resolution of FTIR methodology precludes a more detailed analysis at the conformational level. We previously described a combined NMR-FTIR study of the intermediate using the construct PSD95-PDZ3^302–402^ (Fig. 1B). This construct possesses an experimental advantage, which is the slowest misfolding of the whole PDZ collection. Thus, the intermediate that mainly populates around 60 °C will remain mostly unaltered in solution during time sufficient for experimental measurements. HSQC-NMR experiments^26^ showed that the region around the strands β1 and β5, which organize a short antiparallel hairpin (Fig. 1), roughly maintains its structural arrangement in the intermediate. In contrast, the sheet organized by strands β2, β3 and β4 might be disorganized or rearranged to some extent, although some of the contacts between β2 and helix α2 still remain. Also, the decrease of helical content upon heating at 60 °C was mainly due to the disorganization of helix α1, whereas the α2-helix chemical shifts remained in the intermediate. Furthermore, ^1^H-^15^N correlation spectra showed that mainly those residues of β1 and β5 strands that make contacts with the helix α2 were conformationally stable in the intermediate. This behaviour is fully compatible with the lowering of the α-helix and β-sheet components upon heating shown by FTIR experiments in this work (Fig. 3).

The intermediate described in the present study is a low energy species that populates upon thermal unfolding. As we mentioned in the Introduction, the high-energy intermediate detected by the Gianni and Jemth groups from kinetic approaches was also conformationally analyzed by Φ-value analysis using the same construct PSD95-PDZ3^302–402^ (Fig. 1B)^27^. It is quite curious that this analysis shows the early folding events are driven by the formation of such an α2 helix, which is consolidated by interactions with regions corresponding to β1 and β2 strands in the native state. Moreover, a molecular dynamics simulation using these Φ-values as restraints, showed that regions corresponding to the native secondary structure elements α2 and β5 are relatively well structured in the folding transition state. This conformational arrangement is compatible with the one detected by HSQC-NMR at 60 °C for the equilibrium intermediate.

On the other hand, the conformational analysis of misfolding shows that the examples with the strict PDZ fold behave the same, aggregating in the form of worm-like fibrils, whereas the extra-element of nNOS-PDZ favours competition between amorphous aggregation *versus* fibrilation, while that of PSD95-PDZ3^302–402^ decreases the population of the intermediate and slows down misfolding. Moreover, the occurrence of swapped structures in ZO2-PDZ2 completely precludes the organization into fibrils. Thus, the inclusion of extra-elements essentially modulates the misfolding behaviour by preventing it to some extent, whereas the swapping event clearly prevents misfolding. Taken together, we can observe that all these natural features may have, among others, a common purpose, which is to contribute to the evolutionary pressure to avoid misfolding and aggregation in these domains that, according to our results, are naturally prone to generate equilibrium intermediate states and supramacromolecular structures. More experiments in this regard are always welcome.

## Methods

The expression-optimized DNA sequences of Erbin-PDZ (given generously by Prof. Sachdev Sidhu, University of Toronto, Canada), PSD95-PDZ2 (including residues 155 to 249 in PSD95 numbering), nNOS-PDZ (including residues 1 to 127 in nNOS numbering), ZO2-PDZ2 (including residues 306 to 385 in ZO2 numbering), and PSD95-PDZ3^302–415^ (including residues 302 to 415 in PSD95 numbering), were sub-cloned into pBAT4 vector (EMBL Core Purification Facility) and expressed in *Escherichia coli BL21/DE3* cells. Protein purification was carried out as previously described^17^. We obtained around 15 mg of highly pure protein per liter of LB culture. MALDI-TOF experiments achieved at the Scientific Instrumentation Services (CIC) of the University of Granada confirmed the molecular mass of 11182 Da for Erbin-PDZ, 9968 Da for PSD95-PDZ2, 13534 Da for nNOS-PDZ, 9083 Da for ZO2-PDZ2 and 13466 Da for PSD95-PDZ3^302–415^ and purity of the protein. The extinction coefficients were 1425 cm^−1^·M^−1^ for Erbin-PDZ, 4335 cm^−1^·M^−1^ for PSD95-PDZ2, 1240 cm^−1^·M^−1^ for nNOS-PDZ, 1627 cm^−1^·M^−1^ for ZO2-PDZ2 and 2985 cm^−1^·M^−1^ for PSD95-PDZ3^302–415^, determined as described^28^, at 278 nm. Experimental samples were always prepared by extensive dialysis against 50 mM potassium phosphate buffer at pH 7.5 and 4 °C.

Differential scanning calorimetry (DSC) experiments were performed in a VP-DSC instrument from Microcal INC. as described previously. Fourier transform infrared spectroscopy (FTIR) and Thioflavine T (ThT) fluorescence growth curves experimental details were also carried out as it is referred in previous references^8^,^26^.

## Additional Information

**How to cite this article**: Murciano-Calles, J. *et al.* Common features in the unfolding and misfolding of PDZ domains and beyond: the modulatory effect of domain swapping and extra-elements. *Sci. Rep.* **6**, 19242; doi: 10.1038/srep19242 (2016).

## Supplementary Material

Supplementary Information
