# Supplementary material for: Common features in the unfolding and misfolding of PDZ domains and beyond: the modulatory effect of domain swapping and extra-elements
Source: Sci Rep. 2016 Jan 12;6:19242. doi: 10.1038/srep19242 (PMC4709687; doi:10.1038/srep19242)
Supplement: Supplementary Information [file srep19242-s1.pdf]

Supplementary Information for Publication

Common features in the unfolding and misfolding of PDZ domains and beyond: the modulatory effect of domain swapping and extra-elements

Javier Murciano-Calles [§,†,*], *Jofre Güell-Bosch* [¶], *Sandra Villegas* [¶], *Jose C. Martinez* [§,*]

[§] Departmento de Química Física e Instituto de Biotecnología, Facultad de Ciencias, Universidad de Granada, 18071, Granada, Spain.

[¶] Department de Bioquímica I Biologia Molecular, Facultat de Biociències, Universitat Autònoma de Barcelona, Bellaterra 08193, Barcelona, Spain.

MATHEMATICAL DESCRIPTION OF THE DSC MODEL AND OF THE FITTING PROCEDURE

Among the variety of biophysical techniques available, differential scanning calorimetry would seem to be the best choice to detect any equilibrium conformation(s) that might be populated during unfolding. The two unfolding transitions observed for Erbin-PDZ and PSD95-PDZ2 in Fig. 2 over all protein concentrations undoubtedly indicate the existence of not less than three well defined macroscopic states during unfolding. Both endotherms distance themselves from each other along the T-axis concomitantly with an increase in protein concentration, suggesting some degree of association of the intermediate state. The effect of protein concentration on DSC traces has been described elsewhere in some other examples and is a consequence of the increase in population of the associated species concomitant with protein concentration [1,2]. Thus, at the low PDZ concentrations used in thermal unfolding experiments the associated intermediate populates in a lesser extent (Supplementary Figure S1). This behaviour is shown in Fig. 2, where the behaviour of the DSC trace at 40-50 μM is more that of a single-transition than those obtained at higher protein concentrations, where both endothermic effects are well distinguished.

The three-state model including two equilibriums with an oligomeric intermediate ($N \rightleftarrows (1/n)I_n \rightleftarrows U$), initially used to analyze the calorimetric traces has been previously described [3]. The more complex three-state model displaying three equilibriums presents three conformational states plus the self-association of one of them in equilibrium. Thus, if we consider that the intermediate state self-associates with a certain n stoichiometry, we obtain the following scheme:

$$\begin{array}{c} N \rightleftarrows I \rightleftarrows U \\ \upharpoonleft\downharpoonright \\ 1/n\ I_n \end{array} \quad (1)$$

Thus, there are three equilibrium processes: the first one corresponding to N ⇄ I, with a $K_{N\text{-}I}$ equilibrium constant; the second one to the complete domain unfolding, I ⇄ U, whose equilibrium constant is $K_{I\text{-}U,}$ and the third one, to the association equilibrium I ⇄ (1/n) I_n, whose equilibrium constant is defined by $K_{I\text{-}In}$.

The populations of the native, intermediate, oligomeric intermediate and unfolded states (named as f_N, f_I, f_{In} y f_U respectively), can be calculated as a function of the total protein concentration, P, by means of the following expressions:

$$\begin{aligned} f_N &= \frac{[N]}{P} &\rightarrow\ & [N] = f_N \cdot P \\ f_U &= \frac{[U]}{P} &\rightarrow\ & U = f_U \cdot P \\ f_I &= \frac{[I]}{P} &\rightarrow\ & [I] = f_I \cdot P \\ f_{In} &= \frac{n[I_n]}{P} &\rightarrow\ & [I_n] = f_{In} \cdot \frac{P}{n} \end{aligned} \quad (2)$$

Because in every moment the sum of the populations of the conformational states has to be equal to the unit ($f_N + f_I + f_U + f_{In} = 1$), the next equation can be easily deduced:

$$[I_n] = (1 - f_N - f_U - f_I) \cdot \frac{P}{n} \quad (3)$$

The equilibrium constants can be defined as a function of the populations as follows:

$$K_{N-I} = \frac{[I]}{[N]} = \frac{f_I}{f_N}$$

$$K_{I-U} = \frac{[U]}{[I]} = \frac{f_U}{f_I} \tag{4}$$

$$K_{I-In} = \frac{[I_n]^{1/n}}{[I]}$$

By replacing Eq. 3 in the last expression, we obtain:

$$K_{I-In} = \frac{[I_n]^{1/n}}{[I]} = \frac{\left(\frac{f_{In} \cdot P}{n}\right)^{(1/n)}}{f_I \cdot P} \quad \rightarrow \quad K_{I-In} = \frac{f_{In}^{1/n} \cdot P^{1/n}}{f_I \cdot n^{1/n} \cdot P} \tag{5}$$

And, by reorganizing this expression, we will find:

$$f_{In}^{1/n} = K_{I-In} \cdot f_I \cdot n^{1/n} \cdot \frac{P}{P^{1/n}} \quad \rightarrow \quad f_{In} = K_{In}^{n} \cdot f_I^{n} \cdot n \cdot P^{n-1} \tag{6}$$

Since $f_{In} = (1 - f_N - f_U - f_I)$ and, taking into account that $f_N = f_I / K_{N\text{-}I}$ and $f_U = K_{I\text{-}U} \cdot f_I$, we can write the next expression as a function of the unique unknown magnitude, f_I:

$$n \cdot K_{I-In}{}^{n} \cdot K_{N-I} \cdot P^{n-1} \cdot f_I^{n} + (1 + K_{N-I} + K_{N-I} \cdot K_{I-U}) \cdot f_I - K_{N-I} = 0 \tag{7}$$

By solving Eq. 7 it is possible to deduce the thermodynamic information of the system. In order to obtain the value of f_I we have applied a numerical approach, such as Newton´s method, used to solve potential equations of the general formula $g(x_n)=0$. This is an iterative method, where the initial estimate (x_n) progressively approaches the correct value according to:

$$x_{n+1} = x_n - \frac{g(x_n)}{g'(x_n)} \tag{8}$$

where x_{n+1} represents the value of the tangent line to the $g(x_n)$ function at x_n. We can confirm that 10 calculations are enough for a precise definition of the f_i value.

To complete the equations of this model it is necessary to define the heat capacities of the four conformational states, always defined as a linear function of temperature:

$C_{pN} = a + b \cdot T$ Native state

$C_{pU} = c + d \cdot T$ Unfolded state

$C_{pIn} = h + i \cdot T$ Oligomeric intermediate state (9)

$C_{pI} = p + q \cdot T$ Intermediate state

To define the enthalpy values for every involved process, the reference temperatures $T_{N\text{-}I}$, $T_{I\text{-}U}$ and $T_{I\text{-}In}$, related to the enthalpy values $\Delta H_{N\text{-}I}(T_{N\text{-}I})$, $\Delta H_{I\text{-}U}(T_{I\text{-}U})$ y $\Delta H_{I\text{-}In}(T_{I\text{-}In})$ are considered:

$$\Delta H_{N-I} = \Delta H_{N-I}(T_{N-I}) + ((p-a)\cdot(T - T_{N-I})) + \left(\frac{(q-b)\cdot(T^2 - T_I^2)}{2}\right)$$
$$\Delta H_{I-U} = \Delta H_{I-U}(T_{I-U}) + ((p-c)\cdot(T - T_{I-U})) + \left(\frac{(q-d)\cdot(T^2 - T_{I-U}^2)}{2}\right) \quad (10)$$
$$\Delta H_{I-In} = \Delta H_{I-In}(T_{I-In}) + ((h-p)\cdot(T - T_{I-In})) + \left(\frac{(i-q)\cdot(T^2 - T_{I-In}^2)}{2}\right)$$

In a similar way, changes in entropy can be also estimated, from the relationship

$dS = {dH}/{T} = C_p \cdot {dT}/{T}$ giving place to the next equations:

$$\Delta S_{N-I} = \Delta S_{N-I}(T_{N-I}) + \left((p-a)\cdot \ln\left({T}/{T_{N-I}}\right)\right) + ((q-b)\cdot(T - T_{N-I}))$$
$$\Delta S_{I-U} = \Delta S_{I-U}(T_{I-U}) + \left((p-c)\cdot \ln\left({T}/{T_{I-U}}\right)\right) + ((q-d)\cdot(T - T_{I-U})) \quad (11)$$

$$\Delta S_{I-In} = \Delta S_{I-In}(T_{I-In}) + \left((h-p)\cdot\ln\left(T/T_{I-In}\right)\right) + \left((i-q)\cdot(T-T_{I-In})\right)$$

In this case, $\Delta S_{N\text{-}I}\,(T_{N\text{-}I})$, $\Delta S_{I\text{-}U}\,(T_{I\text{-}U})$ y $\Delta S_{I\text{-}In}\,(T_{I\text{-}In})$ values are defined by:

$$\begin{aligned}
\Delta S_{N-I}(T_{N-I}) &= \frac{\Delta H_{N-I}(T_{N-I})}{T_{N-I}} \\
\Delta S_{I-U}(T_{I-U}) &= \frac{\Delta H_{I-U}(T_{I-U})}{T_{I-U}} \\
\Delta S_{I-In}(T_{I-In}) &= \frac{\Delta H_{I-In}(T_{I-In})}{T_{I-In}} + R\cdot\ln\left(\frac{2\cdot(1/2)^{1/n}\cdot P^{1/n}}{n^{1/n}\cdot P}\right)
\end{aligned} \tag{12}$$

From the known equations ΔG = ΔH-T·ΔS and ΔG = -RTlnK, the values of equilibrium constants and hence the populations can be estimated as a function of temperature. Finally, the global enthalpy change of the unfolding process is expressed by the sum of each of the enthalpies of the different state, weighted by its respective population:

$$\Delta H = \Delta H_{N-I}\cdot f_I + (\Delta H_{N-I} + \Delta H_{I-U})\cdot f_U + (\Delta H_{I-U} + \Delta H_{I-In})\cdot f_{In} \tag{13}$$

The global heat capacity of the process, which is measured experimentally, corresponds to the heat capacity of the native state plus the enthalpy dependence on the temperature upon unfolding, as indicated by the relation $C_p = C_{pN} + d\Delta H/dT$. Such function has been used to achieve the non-linear analysis of C_p *vs* T experimental data.

For the analysis of the DSC traces of Erbin-PDZ and PSD95-PDZ2 shown in Figure 2 and obtained at different protein concentrations we took it, as is assumed by the model, that a single set of thermodynamic magnitudes should be compatible with all the experimental traces, their differences being a consequence of the changes in equilibrium induced by variations in protein

concentration. Thus we undertook an overall analysis to obtain the same heat-capacity $C_{pN}(T)$, $C_{pU}(T)$, $C_{pI}(T)$ and $C_{pIn}(T)$ functions (Eq. 9) for the five experiments, as well as the $T_{I\text{-}In}$ and $\Delta H_{I\text{-}In}(T_{I\text{-}In})$ parameters related to the association-dissociation process. The $T_{N\text{-}I}$, $T_{I\text{-}U}$, $\Delta H_{N\text{-}I}(T_{N\text{-}I})$, $\Delta H_{I\text{-}U}(T_{I\text{-}U})$, $\Delta H_{I\text{-}In}(T_{I\text{-}In})$ and parameters were fitted individually for each experiment in an attempt to assess the standard error associated to this value from the average. Supplementary Table S1 shows the values, including the errors, for all the thermodynamic magnitudes. Calculations were made using Sigma Plot 2000 (Systat Software Inc.).

[1] Filimonov, V. V. and V. V. Rogov, Reversible association of the equilibrium unfolding intermediate of lambda Cro repressor, Journal of Molecular Biololgy, 255 (1996) 767-777.
[2] Ruiz-Sanz, J., V. V. Filimonov, E. Christodoulou, C. E. Vorgias, and P. L. Mateo, Thermodynamic analysis of the unfolding and stability of the dimeric DNA-binding protein HU from the hyperthermophilic eubacterium Thermotoga maritima and its E34D mutant, European Journal of Biochemistry, 271 (2004) 1497-1507.
[3] J. Murciano-Calles, E.S. Cobos, P.L. Mateo, A. Camara-Artigas, J.C. Martinez, A comparative analysis of the folding and misfolding pathways of the third PDZ domain of PSD95 investigated under different pH conditions, Biophysical Chemistry, 158 (2011) 104-110.

Table S1. Thermodynamic parameters of the thermal unfolding of PSD95-PDZ3$^{302\text{-}392*}$, Erbin-PDZ and PSD95-PDZ2 in 50 mM potassium phosphate pH 7.5 obtained from the analysis of DSC experiments (Figure 2).

PDZ domain	$T_{N\text{-}I}$ (ºC)	$\Delta H_{N\text{-}I}(T_{N\text{-}I})$ $kJ\cdot mol^{-1}$	$\Delta G_{N\text{-}I}(298)$ $kJ\cdot mol^{-1}$	$T_{I\text{-}U}$ (ºC)	$\Delta H_{I\text{-}U}(T_{I\text{-}U})$ $kJ\cdot mol^{-1}$	$\Delta G_{I\text{-}U}(298)$ $kJ\cdot mol^{-1}$	$T_{I\text{-}In}{}^{\#}$ (ºC)	$\Delta H_{I\text{-}In}(T_{I\text{-}In})^{\#}$ $kJ\cdot mol^{-1}$	$\Delta G_{I\text{-}In}(343)^{\#}$ $kJ\cdot mol^{-1}$
**PSD95-PDZ3$^{302\text{-}392}$**	52 ± 3	250 ± 20	28 ± 8	79 ± 3	215 ± 10	17 ± 6	125 ± 4	-135 ± 15	-27 ± 5
**Erbin-PDZ**	63 ± 2	391 ± 16	61 ± 7	109 ± 1	165 ± 16	26 ± 5	126 ± 3	-58 ± 7	-20 ± 4
**PSD95-PDZ2**	61 ± 1	312 ± 4	42 ± 5	78 ± 1	157 ± 4	27 ± 2	135 ± 1	-153 ± 1	-19 ± 4

* Taken from Murciano-Calles J., Martinez J.C., Marin-Argany M., Villegas S., Cobos E.S. (2014) A thermodynamic study of the third PDZ domain of MAGUK neuronal protein PSD-95 reveals a complex three-state folding behaviour. *Biophysical Chemistry 185 1-7*.

# Since the analysis is done including several traces, which are obtained at different concentrations of protein, the concentration-dependent thermodynamic magnitudes of the association equilibrium I ⇄ (1/n) In were calculated at a reference concentration of 100 µM.

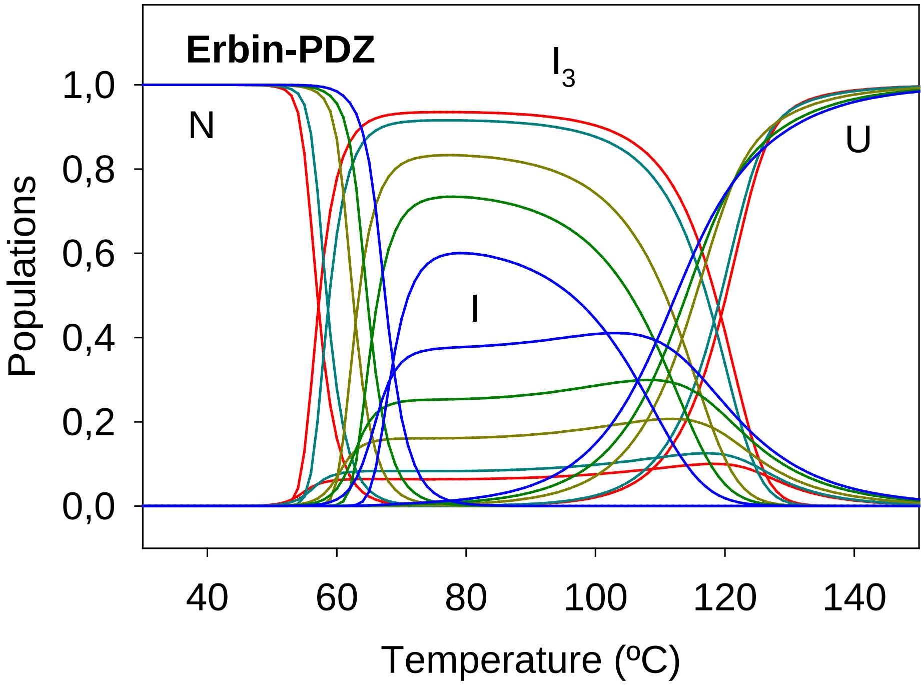

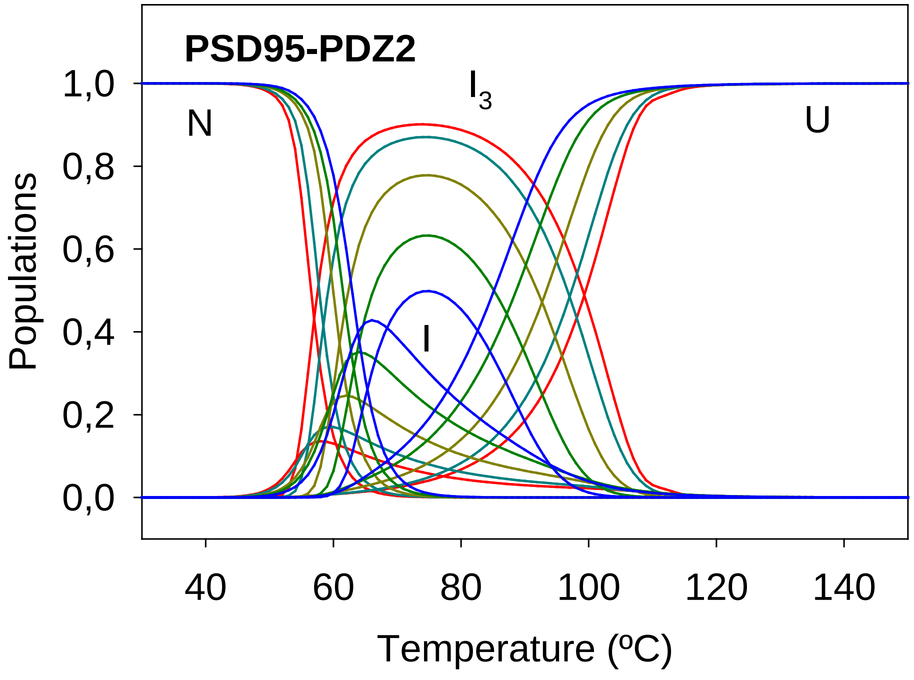

Figure S1. Distribution of populations of the different PDZ states for the different protein concentrations assayed by DSC. The colour code is the same than that of Figure 2. An increase on I_3-state population can be seen on protein concentration.

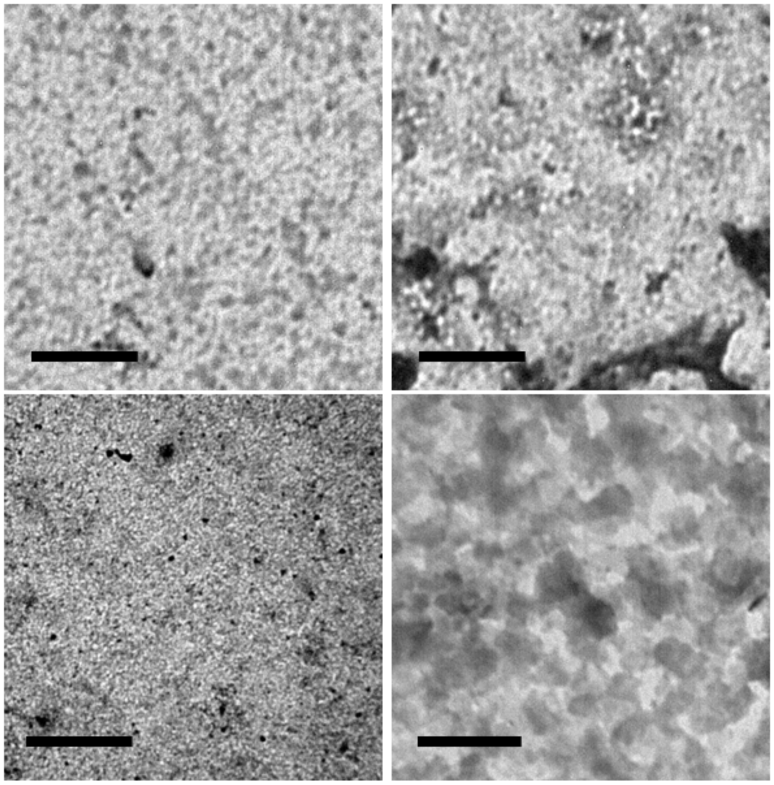

Figure S2. Transmission electron microscopy (TEM) analysis after incubation of the different PDZ domains at 60 ºC during 24 hours. From left to right and from top to bottom: Erbin-PDZ, PSD95-PDZ2, nNOS-PDZ and ZO2-PDZ2. The horizontal black bar corresponds with a length of 200 nm.

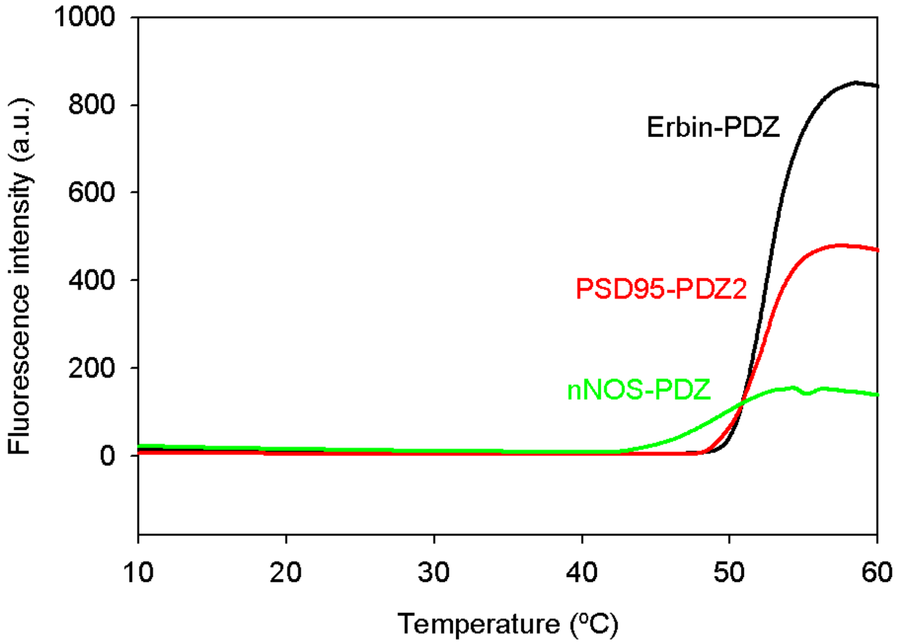

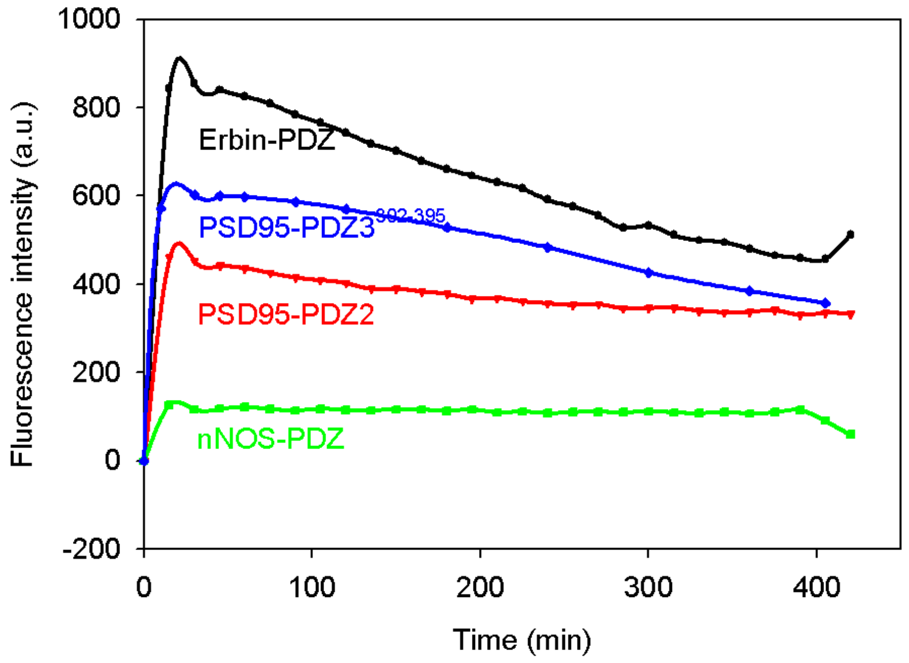

Figure S3. Growth kinetics of ThT fluorescence emission. 8 mg·mL^{-1} PDZ samples (3 mg·mL^{-1} in the case of nNOS-PDZ) were incubated in the presence of 12 µM of ThT at pH 7.5. The upper panel shows the temperature dependence of fluorescence emission at 485 nm. In the lower panel the time dependence of fluorescence signal versus incubation time at 60 ºC is plotted.

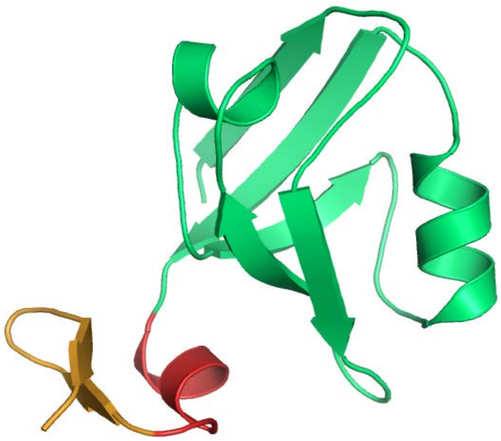

β-1 β-2 β-3 α-1 β-4

PSD95-PDZ3[302-415] -MGLG-EEDIPREP RRIVI-HR GSTGL **G**FN**I**VGGE DG EG**I**F**IS**FIL AGG P**A**DL SGE**LR**K **GD**Q**I**

β-4 α-2 β-5 α-3 β-6 β-7

PSD95-PDZ3[302-415] **L**SV**N**GV DLR**N**A SHE**QA**AIA**LK**N AG QTVT**I**IAQY K P**E**EYSRFE AKS RVNS SG RIVTN

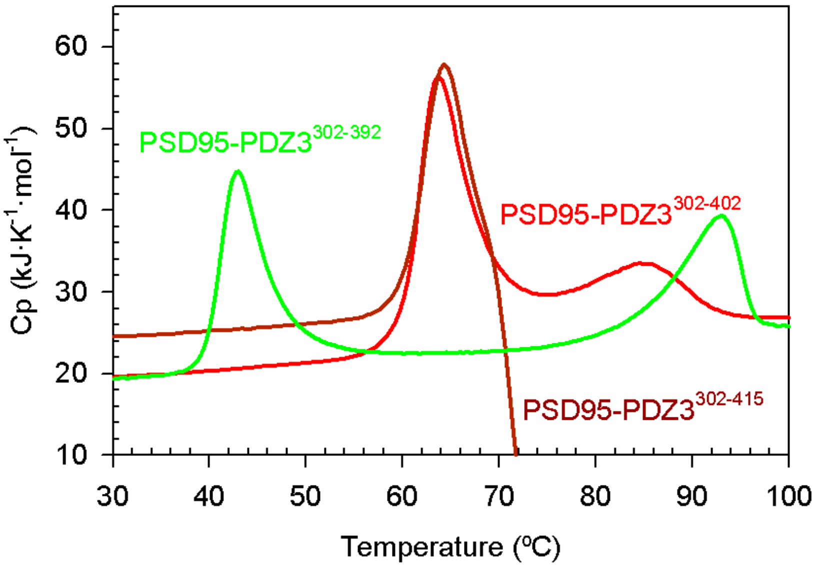

Figure S4. A comparison between different PSD95-PDZ3 constructs. The X-ray structure of the longer construct is shown at the top (PDB Code: 1BE9), followed by a distribution of the secondary structure elements within the sequence. The graph below collects the DSC profiles obtained for the three constructs at 1.5 mg·mL^{-1} in 50 mM K-phosphate buffer pH 7.5. The colour code is kept in all panels.
